# Visual masking and the dynamics of human perception, cognition, and consciousness A century of progress, a contemporary synthesis, and future directions

**DOI:** 10.2478/v10053-008-0009-0

**Published:** 2008-07-15

**Authors:** Ulrich Ansorge, Gregory Francis, Michael H. Herzog, Haluk Öğmen

**Affiliations:** 1 Abteilung für Psychologie und Sportwissenschaft, Universität Bielefeld, Germany; 2 Department of Psychological Sciences, Purdue University, USA; 3 Laboratory of Psychophysics, Brain Mind Institute, École Polytechnique Fédérale de Lausanne, Switzerland; 4 Center for Neuro-Engineering and Cognitive Science, Department of Electrical & Computer Engineering, Houston, USA

**Keywords:** backward masking, dynamic vision, modeling

## Abstract

The 1990s, the “decade of the brain,” witnessed major advances in the study of visual perception, cognition, and consciousness. Impressive techniques in neurophysiology, neuroanatomy, neuropsychology, electrophysiology, psychophysics and brain-imaging were developed to address how the nervous system transforms and represents visual inputs. Many of these advances have dealt with the steady-state properties of processing. To complement this “steady-state approach,” more recent research emphasized the importance of dynamic aspects of visual processing. Visual masking has been a paradigm of choice for more than a century when it comes to the study of dynamic vision. A recent workshop (http://lpsy.epfl.ch/VMworkshop/), held in Delmenhorst, Germany, brought together an international group of researchers to present state-of-the-art research on dynamic visual processing with a focus on visual masking. This special issue presents peer-reviewed contributions by the workshop participants and provides a contemporary synthesis of how visual masking can inform the dynamics of human perception, cognition, and consciousness.

## THE DYNAMICS OF INFORMATION PROCESSING

The 1990s, the “decade of the brain,” witnessed major advances in the study of visual perception, cognition, and consciousness. Impressive techniques in neurophysiology, neuroanatomy, neuropsychology, electrophysiology, psychophysics and brain-imaging were developed to address how the nervous system transforms and represents visual inputs. Many of these advances have dealt with the steady-state properties of information processing. To complement this approach, some researchers emphasized the importance of *dynamic* aspects of visual processing.

In June 2006, we brought together a group of researchers interested in the dynamics of visual perception and cognition. The workshop was generously funded by the Volkswagen Research Foundation and the Hanse-Wissenschafts Kolleg (HWK), which also provided valuable help in arranging and hosting the workshop on the HWK campus in Delmenhorst, Germany. We welcomed researchers from Estonia, France, Germany, Israel, Lithuania, Poland, Sweden, Switzerland, the UK, and the US. This special issue gives an overview of the topics covered on this workshop.

We focused on one of the most important subfields of dynamic visual processing: masking effects. Under masking conditions, the percept of a briefly flashed visual target is often weakened when a masking stimulus is presented in spatial and temporal proximity. If the target were presented by itself, it would be clearly seen (Bachmann, 1994; Breitmeyer & Öğmen, 2006; Stigler, 1910; Werner, 1935). Masking is an important part of the study of perception and cognition. It is used both to investigate the properties of the visual system and as a tool to isolate many other aspects of cognition. Two applications of masking deserve special notice.

Consciousness research. Masking can systematically control the degree of conscious registration of a stimulus. Thus, masking provides an excellent paradigm to investigate the dynamics of conscious and unconscious processing (e.g., Breitmeyer, Öğmen, & Chen, 2004; Dennett, 1991; Klotz & Neumann, 1999; Lachter, Durgin, & Washington, 2000; Vorberg, Mattler, Heinecke, Schmidt, & Schwarzbach, 2003; for a comparison with other techniques, see Kim & Blake, 2005). Recent work also uses masking techniques to connect conscious awareness to neurophysiological properties (e.g., Aron, Schlaghecken, Fletcher, Bullmore, Eimer, Barker et al., 2004; Eimer & Schlaghecken, 1998; Jaśkowski, Van der Lubbe, Schlotterbeck, & Verleger, 2002; Jaśkowski, Skalska, & Verleger, 2003; Macknik & Martinez-Conde, 2004; Pinel, Rivière, Le Bihan, & Dehaene, 2001; VanRullen & Koch 2003). Importantly, while much research on consciousness involves the study of neurologically impaired patients (e.g. Goodale, Milner, Jakobson, & Carey, 1991; Weiskrantz, Warrington, Sanders, & Marshall, 1974), masking can be used to study the distinction between conscious and unconscious processing in normal observers. The special issue covers research concerning the impact of masked unconscious input on attention (Scharlau, this volume), semantic processing (Kiefer, this volume), and response activation (Ansorge, Neumann, Becker, Kälberer, & Cruse. this volume; Enns & Oriett, this volume; Jaśkowski & Verleger, this volume; Kiesel, Kunde, & Hoffmann, this volume; Schlaghecken, Rowley, Sembi, Simmons, & Whitcomb, this volume; Sumner, this volume).

Correspondingly, single cell recordings and brain imaging techniques combined with visual masking have provided new insights about which brain areas are involved in conscious and unconscious vision (Dehaene, Naccache, Le Clec’ H, Koechlin, Mueller, Dehaene-Lambertz et al., 1998; Macknik, this volume; Pinel et al., 2001; Rolls & Tovée, 1994). This and related work shows that even during the processing of unconscious inputs, large networks can be recruited. Thus, both psychological experiments and brain research on visual masking suggest that unconscious vision plays an important role in human cognition and can be studied in a rigorous way.

Visual processing. Masking has been used to study detailed properties of the visual system itself. The application of masking to visual processing encompasses a broad range of areas including the perception of contour (Francis, this volume), motion (Öğmen, this volume; Otto, this volume), colour, pattern (Herzog, this volume), stimulus brightness (Rudd, this volume), and spatial location (Breitmeyer, this volume).

Given the strong interest in masking and the frequency of its use as a tool for investigating perceptual, cognitive, and neurophysiological systems, it is perhaps surprising to note that there is currently no generally agreed-upon theory of the mechanisms that are involved in producing masking effects. Researchers who use masking as a tool to explore other issues generally have the implicit theory that the mask interrupts processing or interferes with detection of the target properties. However, these ideas are generally not rigorously investigated (usually because the researcher is actually interested in something other than masking per se).

Likewise, even though masking effects have been studied for nearly a century, there remains much debate about which properties of masking are fundamental and which properties reflect parametric variations of common mechanisms. For example, many older studies asked subjects to report the perceived brightness of a target stimulus, while modern studies tend to ask subjects to make some kind of discrimination of a target. While such changes in criterion content are known to produce quantitative changes in the data, whether these quantitative changes reflect fundamental differences in the underlying processes remains largely unknown.

Correspondingly, the study and use of masking is often hampered by variability of methods, such as divergent stimuli and experimental tasks that are used by different laboratories. Without time-consuming replications and comparisons among these differences, nobody can tell with certainty whether the procedural differences matter.

## THE WORKSHOP GOALS

The goal of the proposed workshop was to bring together researchers who are interested in understanding the mechanisms that produce the many different types and effects of masking. Through intense interaction between groups and researchers, we hoped to gain new insights into current research and identify ideas and methods that would lead to an improved understanding of the role and mechanisms of masking effects at both the behavioural and neurophysiological levels. Because masking plays an integral part in the study of many aspects of cognition, the outcome of the meeting promised to provide new insights into many different areas of human cognition, especially studies of consciousness. To achieve these goals, we invited speakers from a variety of backgrounds.

In addition to sharing state-of-the-art research, we asked the participants to make connections across different domains, identify a framework for discussing visual masking and related topics, raise general questions about the topic, and to promote theoretical speculations. We wanted to look for possible connections and big unanswered questions, and to think about what types of research would really change how researchers consider or use masking techniques, dynamic vision, and consciousness. Hence, the current proceeding was aimed to be a comprehensive and controversial overview of the field rather than a collection of loosely related empirical results.

The contributions to this special issue are based on the presentations at the workshop. The articles are organized in roughly the order of presentation at the workshop (with poster presentations interleaved as appropriate). The contributions do not really need individual introductions, so we chose to use this opening article to describe the main discussions of the workshop itself. The discussions were lively and engaging, and the themes that dominated them are reflected in many of the articles in this special issue.

### A contemporary synthesis of masking research and establishment of future directions

One hope of the workshop was that it might be possible to identify a set of standards for masking experiments and then develop a worldwide accessible data bank that lists the properties of masking for many different circumstances. This kind of rapid data sharing would promote the use and knowledge of masking for both researchers interested in the details of masking *per se* and researchers interested in using masking as a tool to explore other aspects of cognition and consciousness. Discussions on this topic were quite vigorous. On the one hand, there are already *de facto* standards in the mask priming community, who tend to use a relatively narrow set of stimuli (see the papers by Enns & Oriett, this volume; Jaśkowski & Verleger, this volume; Schlaghecken et al., this volume; Sumner, this volume). Such standards are needed to insure that prime stimuli are truly masked. In contrast, some researchers who use masking to study perception were concerned that such standards would inhibit the development of novel experimental paradigms. Although the proposal was not intended to limit research plans, objections to standardization were so strong that there seemed to be no way to structure a database of this sort.

One notable proposal for future directions was a call for a functional perspective on masking research. On the one hand, masking has often been studied as a phenomenon and has been used to study other aspects of perception and cognition. Such work generates valuable data about the properties of masking, but does not always clarify the fundamental basis for these properties. Surely the visual system did not evolve to demonstrate properties of masking; rather the properties of masking must be derived from fundamental ecological solutions to problems that the visual system encounters in a complex spatio-temporal world. Several of the papers in this special issue do use a functional perspective on masking. The contribution of Reeves (this volume) claims that explanations of masking *per se* are of rather low interest if there are no functional aspects involved. Likewise, Öğmen (this volume) argues that the mechanisms involved in many masking phenomena are fundamentally related to motion deblurring, and Ansorge et al. (this volume) considers the possibility of masking as an inevitable consequence of a visual system validating visual inputs by motor consequences before conscious representation of these inputs.

### Synthesize current and identify future neurophysiological studies

Neurophysiological studies of masking go back many years, but recent developments in technology have greatly increased the study of masking effects and their significance. However, there are now so many different ways to explore brain mechanisms and so many possible properties of neural activity to explore that it is not obvious which techniques and properties are most likely to reveal important information about the neural mechanisms involved in masking. We asked the workshop participants to identify what kinds of neurophysiological studies are most likely to help identify the mechanisms underlying masking effects.

The technologies discussed focused on single cell recordings (Macknik, this volume), functional Magnetic Resonance Imaging (fMRI; Macknik, this volume), transcranial magnetic stimulation (TMS; Kammer, this volume), and Event-Related Potential (ERP; Polat, this volume; Verleger & Jaśkowski, this volume) studies. A common observation was that different conclusions about the effect, location, properties, and influence of masking were reached by different studies and techniques. Variations in stimuli, species, tasks, and contexts make comparisons across studies quite difficult. Even worse, it may be difficult to make precise statements about the neurophysiological underpinnings of masking because it may take place at many different loci and with many different mechanisms.

Single cell recording studies demonstrate this difficulty. Stephen Macknik and Uri Polat summarized their recent single-cell recording investigations of masking. Whereas Macknik found that masking effects reduced responses to a target, Polat found that stimulus activity increased under masking conditions. It is not clear how to reconcile the differences, although there are many candidates and using a common type of stimulus might help.

fMRI techniques for either using or studying masking are still being developed. Thus, it is not surprising that, again, the results do not give a consistent story. Whereas a recent study by John-Dylan Haynes and co-workers (Haynes, Driver, & Rees, 2005) found that the bold fMRI signal correlated with conscious awareness in areas beyond V1, fMRI and single-cell recording studies by Macknik found bold fMRI signals affected in V1 or earlier (cf. Macknik, this volume).

Despite these controversies, it was generally acknowledged that metacontrast masking provides an advantageous experimental procedure for the study of the neural correlates of consciousness with fMRI because the (u-shaped) masking function is able to rule out a variety of candidate brain areas. In essence, one can scan the brain for u-shaped activity functions, because the activity marker of conscious perception (reflected in the masking function) is correlated in a predictable but non-linear (and, hence, nontrivial) manner with a specific independent variable (SOA, Stimulus Onset Asynchrony; or ISI, Inter Stimulus Interval).

TMS studies are also of great interest to masking research because the TMS pulse seems to act somewhat like a mask. On the other hand, there is not yet an adequate understanding of the functional influence of TMS because some results have failed to replicate (Kammer, this volume).

ERP methods are the most common neurophysiological techniques used to study masking (Verleger & Jaśkowski, this volume; Polat, this volume). Here too, though, there are difficulties interpreting data. For instance, shifts of visual attention produce changes in the stimulus-contingent lateralized ERP signal that are not easily discerned from brain activity differences between the hemispheres. The ERP technique has the required temporal resolution to study masking, yet it suffers from a relatively poor spatial resolution.

In general, the discussion about neurophysiological studies made it clear that these methods are less well understood than sometimes claimed or thought. Therefore, caution should be applied if any conclusions are drawn from the physiological results, as long as there is no converging behavioural and psychophysical evidence to support the conclusions. One cannot deny, however, that the neurophysiological methods have great promise for investigating and utilizing masking phenomena.

### Identify future ways to study consciousness

Similar to the development of neurophysiological studies, the study of consciousness has dramatically improved in recent years with new techniques and theories. The properties of masking have contributed substantially to these developments, but perhaps have not been used to their full potential. The meeting attendees were asked to consider how masking effects can best be used to study issues of consciousness in the future.

One important discussion concerned the refinement of measures of residual perception of a masked prime (or test stimulus) in masked priming studies of unconscious visual faculties. In general, if the measure of conscious prime perception in such studies is not exhaustive, there is always the danger that performance in target response tasks is falsely attributed to unconscious visual processing of the masked prime. The most exhaustive procedure of residual conscious prime perception uses the same stimuli, responses, and response mappings in the prime discrimination task as in the target response task, but asks participants to discriminate the masked prime in the prime discrimination task instead of responding to the visible target. Chance performance (d-prime equal to zero) in such an exhaustive prime discrimination task has become somewhat of a standard prerequisite for establishing unconscious vision (e.g., Kiefer, this volume).

At the workshop, this standard was criticized and alternative measures for future masked priming research were suggested (Wiens, this volume). Volker Franz argued that effect sizes of binary decisions reflecting prime discrimination performance should be compared to measures employing the same metric in the target response task. Franz further argued that information-theoretic measurements provided a better analysis of performance. Such measurements have subtle issues, though. For example, a small negative *d*-prime value is usually interpreted as an indication that the observer cannot distinguish the target present and absent cases. However, from an information theoretic point of view, the negative d-prime (no matter how small) indicates that some information was present. If this information can be connected to the priming effect, then a statistically non-significant d-prime may not indicate the absence of conscious awareness of the prime.

In a similar vein, Thomas Schmidt (this volume) proposed an entirely different approach for masked priming. Instead of rendering a stimulus non-conscious, he suggested varying prime-target SOAs to measure both masked priming effects and prime discrimination performance. If it turns out that some variables (e.g., duration of the prime, direction of attention, etc.) have different effects on SOA priming functions than on masking functions, this would imply that SOA priming functions must reflect influences besides those of conscious visual faculties (as the latter are reflected in the masking function).

There were also lively discussions about phenomenal experiences (i.e., qualia, the way things appear in consciousness). Breitmeyer noted that phenomonologically a target may be invisible, but still produce a high d-primemeasure. An example of this is in feature inheritance effects where the target is invisible, but some of its features are visible in the mask (Herzog, this volume; Otto, this volume; for modelling, see Hamker, this volume). Ulrich Ansorge pointed out that these types of phenomena link some masking paradigms to mask priming effects. A particularly important phenomenological experience is that of time itself. In some situations properties of a target are modified in perceived time and order as well as in spatial appearance (cf. Scharlau, this volume).

Finally, it was noted that there is a good chance that conclusions from different experimental masking paradigms and across studies of healthy participants and neuropsychologically impaired subjects could be directly compared with controlled procedures (Breitmeyer, this volume).

### Model development

To promote model development we asked the workshop participants to consider several important issues. First, what are the similarities and differences in models? Are they compatible with one another? Second, what kinds of experiments would provide definitive tests of the models, or of an entire class of models? Third, what are the key problems with existing models? Fourth, what is needed to make the models more applicable for research of cognition in general and for understanding the relationship between masking and cognition?

A wide variety of models were discussed at the workshop. These varied from small sets of differential equations (Hermens & Ernst, this volume), large quantitative simulations of complex interactions among various neural systems (Francis, this volume; Hamker, this volume; Öğmen, this volume), feedforward models (VanRullen, this volume) and processing models that hypothesized interactions but did not include quantitative calculations (Bachmann, this volume; Enns & Oriett, this volume). Many of the quantitative models can be compared to each other, but it is less clear how to compare the quantitative and non-quantitative models.

Greg Francis (this volume) discussed experimental data that are evidence against a wide variety of quantitative models. He argued that a fundamental flaw of all current models is that they one-sidedly focus on the temporal dynamics and do not appropriately take into account processing of specific spatial aspects of test and masking stimuli, such as grouping phenomena. Herzog (this volume) arrives at the very same conclusion from empirical grounds. Pattern as well as metacontrast masking seems to involve complex spatial interactions that may be best explained with spatial perceptual grouping. Still, Bruce Bridgeman (this volume) and Hermens and Ernst (this volume) showed that simple neural network models can simulate many masking effects, including some of these spatial effects. Other discussions emphasized that the modeling work needs to account for task-set influences on processing speed and phenomenally perceived temporal characteristics (e.g., temporal order of stimuli, perceived duration of ISIs, or perceived stimulus duration) under masking conditions (Ansorge et al., this volume).

The discussion revealed that one major difficulty for the development of quantitative models is the computation required to simulate and analyze the models. Simulations of large complex models can take days to complete and much longer to analyze. Moreover, as the simulation increases in complexity, it becomes increasingly difficult to predict the behavior of the model. In many respects, these complex models have to be studied with simulated experiments; in much the same way that empirical research is used to investigate human behavior.

This complexity may be contributing to another aspect of the current state of models: they do not address the same data sets. For example, Rufin VanRullen (this volume) described SpikeNet, which allows for a rapid classification of visual scene images, implying that visual image analysis can escape conscious awareness once awareness is blocked some several tens of milliseconds after the stimulus by a backward mask. At the moment, this model, and the data it explains, seems incompatible with other models of backward masking, and the data they explain. Likewise, none of the models of masking discussed at the workshop make any direct connections to the masked priming literature or related topics such as goal setting.

What became clear from the discussions is that all of the models have their blind spots even if we restrict our analysis to modelling of visual masking, and that therefore one fruitful future research strategy would be to extend each model to the empirical evidence which it currently ignores.

## CONCLUSIONS

In conclusion, the workshop generated a lively discussion and exchange of ideas. The articles in this special issue provide an extended exposition of these contributions. A century of research provides a wealth of information about visual masking, yet we acknowledge that our understanding of masking remains limited. The situation is not very different from other areas of vision, such as form perception. One can draw a “bad news / good news” lesson from this comparison. The bad news is, of course, that our current knowledge does not lead to a simple set of laws or rules that can provide a general understanding of visual masking. The good news is that this failure appears to stem from the fact that masking is not a relatively isolated peculiarity of vision but instead is a complex phenomenon with important implications for many areas of vision science. It involves an extremely broad coverage of visual phenomena including surface, depth, and contour processing, perceptual grouping, attention, contextual effects, awareness, and priming. It has been used to understand many properties of both normal and abnormal visual function. Thus, we expect that our understanding of masking will progress hand in hand with other aspects of visual science with reciprocal and synergetic contributions.

After the workshop, there were a series of further discussions that were carried out through e-mail communications. This included an interesting challenge to the community to predict masking effects. We have archived these discussions (along with some photos of the workshop) at http://lpsy.epfl.ch/VMworkshop/ under the Follow-ups section.
